# Congenital duodenal diaphragm with heterotopic pancreas: a case report and literature review

**DOI:** 10.3389/fped.2025.1590865

**Published:** 2025-05-15

**Authors:** Jinghui Song, Suolin Li, Weili Xu, Chi Sun, Yanbin Fang, Bingzheng Gao, Meng Li

**Affiliations:** Department of Paediatric Surgery, The Second Hospital of Hebei Medical University, Shijiazhuang, China

**Keywords:** congenital duodenal obstruction, heterotopic pancreas, indocyanine green, nasojejunal tube, children

## Abstract

This case report presents a laparoscopic surgery performed at The Second Hospital of Hebei Medical University for a patient with congenital duodenal diaphragm complicated by heterotopic pancreas (HP). The utilization of indocyanine green (ICG) molecular fluorescent imaging technology and the suspension traction method for duodenal obstruction points during the procedure demonstrates the potential for reducing operational complexity and enhancing surgical success rates. Postoperative feeding via a nasojejunal tube is shown to facilitate rapid restoration of enteral nutrition, mitigate postoperative complications, and decrease the duration of hospitalization.

## 1 Introduction

Congenital duodenal obstruction (CDO) is an uncommon congenital gastrointestinal malformation in neonates, occurring in approximately 1.5 per 10,000 live births (1, 2). The duodenal diaphragm, a potential cause of CDO, can result in atresia or stenosis. Currently, surgical intervention remains the sole effective treatment for this condition (3). Heterotopic pancreas (HP) refers to pancreatic tissue situated in an atypical location, lacking anatomical or vascular connections with the orthotopic pancreas. The incidence of HP ranges from 0.5% to 13.7% in autopsy studies (4–6). HP is often asymptomatic and typically discovered incidentally during abdominal surgery (6, 7). In September 2024, our hospital successfully performed a laparoscopic surgery for a congenital duodenal diaphragm with a jejunal HP, utilizing molecular fluorescent imaging with indocyanine green (ICG). This technique, which is rarely reported in the literature, demonstrates significant potential for enhancing surgical precision and patient outcomes. We present our experience to improve surgeons' understanding of congenital duodenal diaphragm with HP and to share surgical and perioperative management strategies.

## 2 Case report

### 2.1 Patient information

A one-day-old female neonate was admitted to our hospital with a primary diagnosis of fetal duodenal obstruction, initially detected during the 25th week of gestation. The infant was delivered via cesarean section at a gestational age of 37^+6^ weeks, weighing 2,500 g. Fetal ultrasonography at 25 weeks gestation revealed a cystic echo in the epigastric region, suggesting duodenal obstruction. At the 29th week of gestation, the family received prenatal counseling at the local hospital, where amniotic fluid karyotype analysis showed no apparent numerical or structural abnormalities. The patient was recommended for referral to our pediatric surgery department for post-natal treatment. The mother continued regular prenatal check-ups until the cesarean section was performed at 37^+6^ weeks of gestation. The infant's vital signs remained stable, with no symptoms of abdominal distension, vomiting, jaundice, or hematochezia. The infant was kept nothing by mouth (NPO), had no bowel movements, and displayed no urinary abnormalities. The infant was admitted with a diagnosis of duodenal obstruction. Clinical examination revealed a distended abdomen with the absence of gastrointestinal patterns or peristaltic waves. There were no abdominal wall varicosities, compression pain, or palpable masses. Murphy's sign was negative, and there was no rebound pain or muscle rigidity. Percussion and auscultation of bowel sounds were unremarkable.

### 2.2 Therapeutic intervention

#### 2.2.1 Preoperative preparation

**Figure 1:**
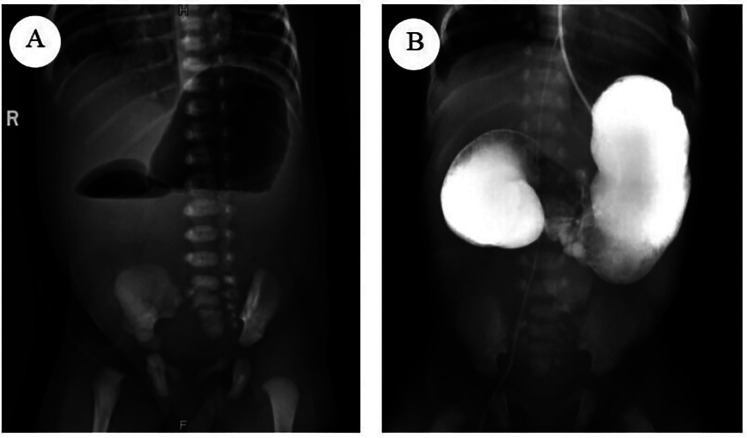
**(A)** The upright abdominal radiograph reveals the characteristic double-bubble sign, indicative of duodenal obstruction. **(B)** The Upper gastrointestinal contrast study revealed an enlarged gastric lumen, with minimal contrast medium entering the duodenum and small intestine.

Upon admission, the patient received level I care and was maintained on NPO status, with gastrointestinal decompression, acid suppression, and parenteral nutrition support. A Peripherally Inserted Central Catheter (PICC) was placed to facilitate long-term intravenous infusion and nutritional support therapy. The patient's overall condition was evaluated through a comprehensive series of diagnostic procedures, including routine blood tests, blood biochemistry, coagulation profile, preoperative screening, cardiac ultrasound, and posteroanterior chest radiograph. The Upright abdominal radiograph reveals the characteristic double-bubble sign, indicative of duodenal obstruction (Figure 1A). An upper gastrointestinal contrast study using iohexol dilution revealed an enlarged gastric lumen, with minimal contrast medium entering the duodenum and small intestine (Figure 1B). Cardiac ultrasound identified patent foramen ovale and patent ductus arteriosus. Abdominal ultrasound showed unobstructed umbilical veins and venous conduits, with no evidence of space-occupying lesions in the liver, gallbladder, pancreas, spleen, or kidneys. Laboratory test results indicated a white blood cell count of 16.1 × 10⁹ cells/L with 74.79% neutrophils and an activated partial thromboplastin time of 90.5 s. Based on these findings, neonatal infections and coagulation disorder were additionally diagnosed. The patient was treated with amoxicillin-clavulanate potassium for infection control, intramuscular vitamin K1, and plasma transfusion to address the coagulation disorder. Following a comprehensive review, no surgical contraindications were identified, and the infant underwent surgery on the fourth day of life. The family was informed of the treatment plan and provided written informed consent.

#### 2.2.2 Surgical intervention

**Figure 2:**
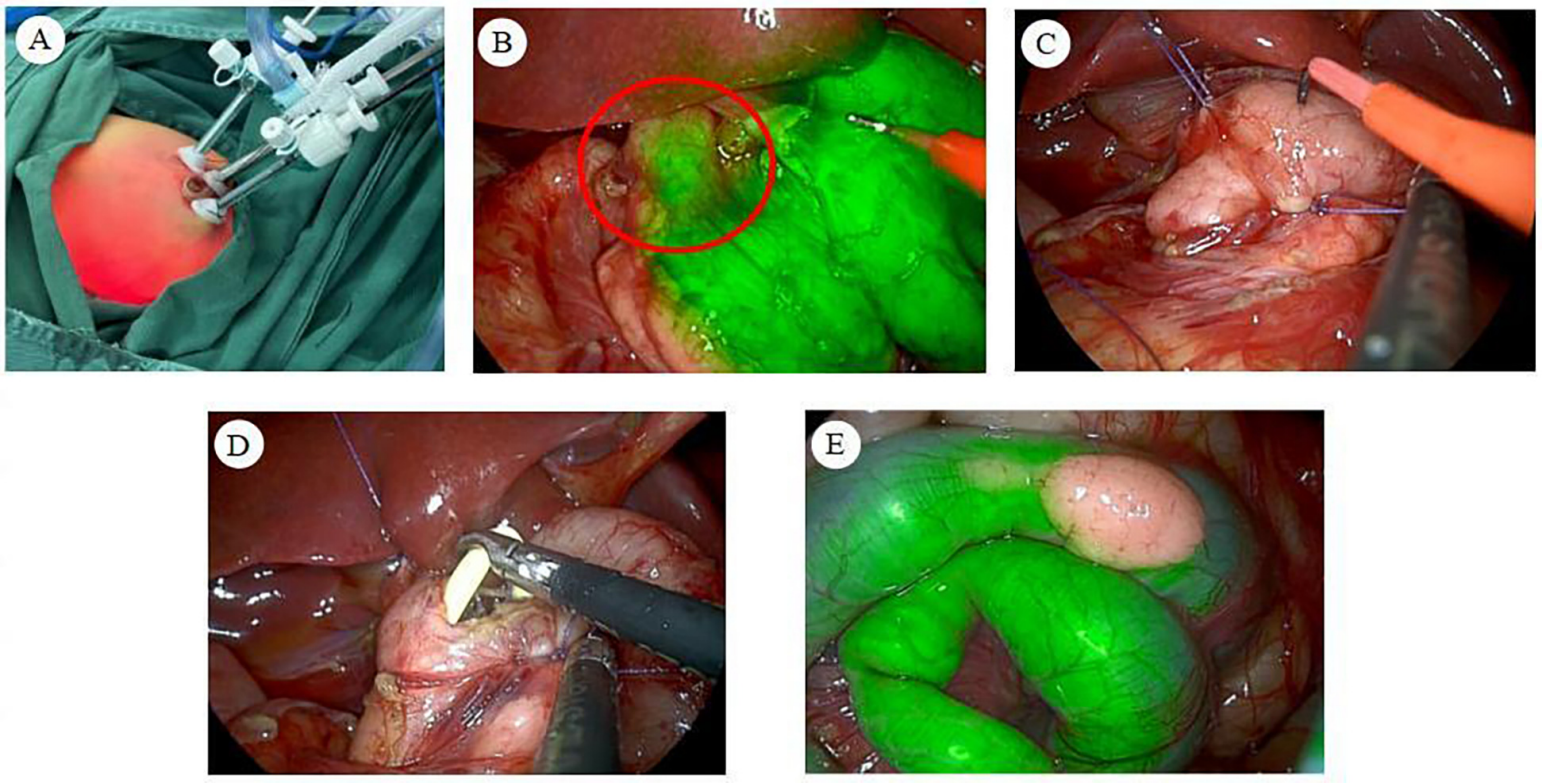
**(A)** Trocar placement in the abdomen. **(B)** Under fluorescence mode, the duodenal segment at the level of the upper border of the pancreas exhibited a sudden narrowing, with no fluorescence visible distal to the obstruction, thereby identifying the obstruction point. **(C)** Suspending the anterior and posterior wall tissues at the duodenal obstruction site allows for optimal exposure and stabilization of the obstruction point. **(D)** A nasojejunal tube is placed intraoperatively, bypassing the intestinal anastomosis and extending into the distal intestine. **(E)** The heterotopic pancreas revealed under ICG molecular fluorescence imaging.

Following successful anesthesia, the infant was positioned supine. The perineum and surrounding area were thoroughly disinfected, and sterile drapes were applied. Subsequently, 5 ml of ICG was administered via the nasogastric tube. A 5 mm arc-shaped incision was made at the lower edge of the umbilicus, and the subcutaneous tissues were carefully dissected. A 5 mm Trocar was inserted under direct vision to establish a CO2 pneumoperitoneum, with the pressure set to 6 mmHg. A 5 mm 30° laparoscope was then introduced. Under laparoscopic guidance, incisions were made on the upper left and lower right borders of the umbilicus. A 3 mm trocar was inserted at each puncture site (Figure 2A).

During the abdominal cavity exploration, it was noted that the gastric pylorus and superior duodenum had lost their normal morphology. The maximal expansion diameter measured approximately 3.5 cm. Ablation electrodes were utilized to divide the hepatoduodenal and gastrocolic ligaments, exposing the pancreas, which appeared morphologically normal. Upon switching to fluorescence mode, a localized obstruction in the duodenum was revealed at the level of the pancreas' superior border, where the intestinal lumen abruptly narrowed (Figure 2B). The distal segment of the obstruction had a diameter of approximately 0.8 cm. The obstruction was attributed to the presence of a duodenal septum, and the following procedure was performed. 5-0 absorbable wire was inserted through the right upper abdominal subcostal margin and the left upper abdominal wall puncture, respectively. The posterior and anterior wall tissues of the stenotic intestinal segment were hooked, completing the suspension traction of the duodenal obstruction point and exposing and fixing the obstruction point (Figure 2C). The duodenum at the obstruction site was then longitudinally incised with an ablation electrode, revealing a diaphragm within the intestinal lumen. This finding was intraoperatively diagnosed as a duodenal diaphragm. The diaphragm was resected using an ablation electrode. The wound at the diaphragm base was closed with 5-0 absorbable sutures for hemostasis.

A nasojejunal tube was inserted transnasally over the incised duodenal obstruction and into the distal duodenal intestinal lumen (Figure 2D). The anterior wall incision of the duodenum was closed using continuous horizontal sutures with 5-0 absorbable sutures, completing the duodenal reconstruction [longitudinal incision and transverse suture (8)]. Following this, 2 ml of ICG and 5 ml of air were introduced through the nasogastric tube, resulting in the observation of sequential fluorescence in the distal duodenum and proximal jejunum of the anastomosis. This confirmed the patency of the intestinal anastomosis and the absence of leakage.

Upon further examination of the jejunum, a HP measuring approximately 1 cm × 0.5 cm was identified on the jejunal surface, opposite the mesenteric margin, approximately 20 cm from the ligament of Treitz (Figure 2E). This intraoperative finding was diagnosed as a HP on the jejunum. After consultation with the patient's family regarding the diagnosis, we recommended proceeding with a heterotopic pancreatectomy, to which they consented. Subsequently, the intra-abdominal exudate was aspirated, and the anastomosis was meticulously inspected, revealing no abnormalities. The umbilical puncture site was then connected to the right abdominal puncture site, and the jejunum containing the HP was externalized through the umbilical incision. The ablation electrode was utilized to resect the HP along with its attached intestinal wall. The incision at the junction of the HP and the intestinal wall was closed transversely. Laparoscopic-assisted heterotopic pancreatectomy was then completed, followed by the removal of the suspension line and laparoscope.

A total of 5 ml of sodium hyaluronate was administered intraperitoneally. A comprehensive count of instruments and dressings was performed to ensure accuracy. The umbilical incision was closed using continuous sutures with 4-0 absorbable material. Closure of the subcutaneous layer was achieved using interrupted 5-0 absorbable sutures, and the left abdominal puncture site was similarly closed with interrupted 5-0 absorbable sutures. The incisions were then secured using medical-grade bioadhesive.

The surgical procedure was successfully completed, with a duration of 120 min. Intraoperative blood loss was minimal at 2 ml, and 32 ml of plasma was administered without any adverse reactions noted. Subsequent to the family's examination of the specimen, it was submitted for pathological analysis. Upon the restoration of spontaneous respiration, the patient was transferred to the post-anesthesia care unit (PACU) for postoperative monitoring.

## 3 Result

**Figure 3:**
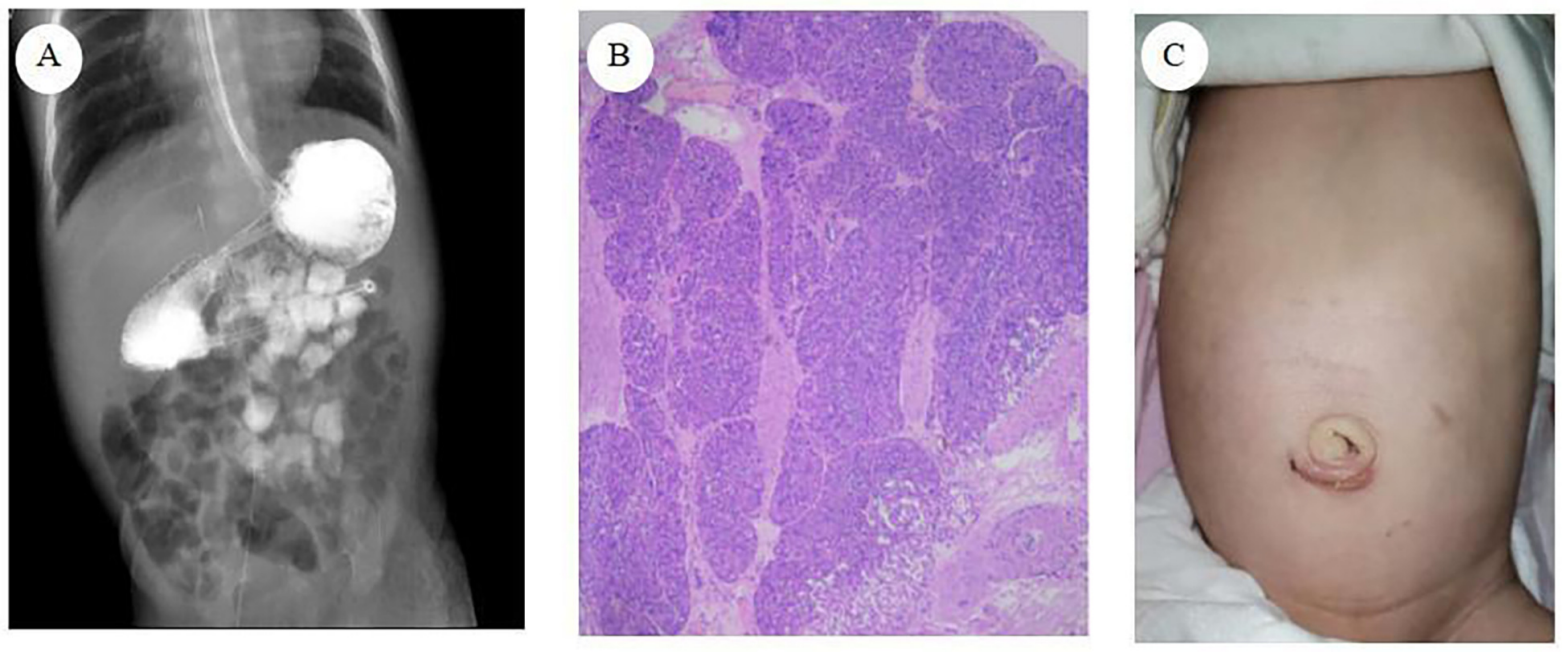
**(A)** Upper gastrointestinal contrast study **(B)** pathological results of heterotopic pancreas tissue **(C)** abdominal wall appearance at one month after surgery.

Postoperatively, the patient received level I care, encompassing continuous electrocardiogram (ECG) and oxygen saturation monitoring, gastrointestinal decompression, and maintenance of a nasojejunal tube. The patient was administered antimicrobial, hemostatic, acid-suppressive, and intravenous nutritional support. On the first postoperative day, the patient passed green stools containing ICG, confirming the restoration of gastrointestinal patency. By postoperative day 3, a 5% glucose solution was administered via the nasojejunal tube without eliciting symptoms of vomiting or abdominal distension. On postoperative day 5, feeding transitioned to hydrolyzed protein formula via the nasojejunal tube. Oral feeding with hydrolyzed protein formula commenced on postoperative day 10, with the feeding volume gradually increased based on the child's tolerance. An upper gastrointestinal contrast study on postoperative revealed proper positioning of the nasojejunal tube, reduced gastric volume, and smooth passage of contrast through the stomach and intestine (Figure 3A). Following a comprehensive review of laboratory test results, including blood routine examination, liver and kidney function, procalcitonin (PCT), and C-reactive protein (CRP), and in the absence of significant abnormalities, the patient was deemed suitable for discharge.

Pathological examination revealed: (1) a duodenal diaphragm exhibiting significant tissue cauterization, minimal chronic mucosal inflammation, and submucosal fibrous tissue hyperplasia; (2) the presence of HP tissue (Figure 3B).

At the 6-month postoperative follow-up evaluation, the infant had been successfully introduced to complementary feeding without subsequent episodes of vomiting, abdominal distension, constipation, inconsolable crying, or milk aversion. The abdominal surgical incision site showed no signs of infection, dehiscence, or other complications. Growth and Development Status: The weight of the child was 7.5 kg, and the length was about 67 cm, both measurements falling within the 25^th^–75th percentiles for nationally established growth standards of infants at this age. Neurodevelopmental evaluations demonstrated age-appropriate attainment of milestones, including the capacity for independent rolling and production of single-syllable vocalizations. The overall postoperative recovery effect of the children was good, and there was no significant difference in growth and development level compared with that of normal term infants.

## 4 Discussion

CDO represents a rare congenital structural anomaly of the gastrointestinal tract in neonates. The etiologies encompass both endogenous causes, such as duodenal atresia, stenosis, and duodenal web, as well as exogenous causes, including annular pancreas, intestinal malrotation, anterior duodenal hilar vein, and gastroduodenal duplication anomalies (9).

Surgical management of congenital duodenal atresia primarily employs two approaches: traditional open surgery and laparoscopically assisted surgery. Following the seminal report by Bax et al. (10) on laparoscopic repair of duodenal atresia (LRDA), laparoscopic-assisted surgical techniques have undergone substantial advancements in the 21st century. The enhanced aesthetic outcome of postoperative incisions has contributed to the increasing preference for this method among both surgeons and families of pediatric patients. A retrospective cohort study by Sidler et al. (11), encompassing 41 children with CDO, demonstrated significant advantages of laparoscopic surgery in postoperative recovery and pain management compared to open surgery. A more recent systematic review and meta-analysis (12), incorporating 11 studies and involving 1,615 pediatric patients, further elucidated the benefits of laparoscopic surgery, revealing reductions in time to first and full feeds, length of hospital stay, and wound infection rates. However, no significant differences were observed between the two approaches regarding anastomotic leakage, anastomotic stenosis, postoperative intestinal obstruction, or overall complication rates.

In this case, the three-port laparoscopic duodenal obstruction point suspension traction method was employed. During the procedure, ICG was administered through a nasogastric tube to facilitate rapid and accurate localization of the duodenal obstruction under fluorescent laparoscopy. This approach addressed the limitations of preoperative ultrasound and upper gastrointestinal contrast studies, which often fail to precisely identify the obstruction point (9). It also aids in determining the endogenous or exogenous origin of the obstruction at an early stage. Following duodenal reconstruction, anastomotic leakage can be readily detected by observing the leakage of a fluorescent agent using ICG molecular fluorescence imaging, applied by exerting pressure on the bowel at both the upper and lower extremities of the anastomosis, thus reducing the risk of postoperative complications. Furthermore, laparoscopic surgery provides a broader field of view and enhanced magnification compared to open surgery. With the assistance of ICG molecular fluorescent imaging technology, the fluorescent background of the intestinal tubes facilitates the identification of this patient's HP located on the jejunum.

Neonatal laparoscopic surgery presents unique challenges, including limited operative space, restricted visual field due to conventional umbilical laparoscope placement, and potential intestinal content spillage during duodenotomy. These factors may compromise procedural efficacy and increase the risk of suture omission and irregular needle spacing during intestinal anastomosis, thereby elevating surgical complexity and postoperative anastomotic leakage rates (13). The technique of suspending the obstructed duodenal segment enhances the exposure of the anastomosis area and maintains bowel stability. This approach not only simplifies surgical steps and reduces the technical complexity of laparoscopic duodenal anastomosis but also minimizes the number of operative incisions, resulting in improved aesthetic outcomes of the abdominal wall postoperatively (Figure 3C). Furthermore, it eliminates the need for intraoperative gastroscopic localization, thus preventing prolonged operative duration and reducing the risk of misidentifying the obstruction point.

Nutritional management plays a crucial role in the postoperative care of infants with CDO. While prolonged parenteral nutrition (PN) maintains essential physiological functions, it poses risks of complications such as infections, cholestasis, and metabolic disturbances. Extended fasting may also lead to intestinal mucosal atrophy and inhibit epithelial cell proliferation, potentially increasing intestinal permeability and facilitating bacterial translocation. In severe cases, this can trigger systemic inflammatory response syndrome (SIRS) (14). Early implementation of enteral nutrition (EN) has shown significant advantages in enhancing nutritional status, promoting wound healing, accelerating intestinal functional recovery, reducing postoperative complications, and supporting catch-up growth in infants.

The prolonged obstruction leads to secondary dilatation of the proximal bowel and thickening of the bowel wall with edema (15). This condition is exacerbated by enteric nervous system hypoplasia and reduced distribution of interstitial cells of Cajal (16), collectively impairing intestinal motility. Postoperative inflammation and edema at the anastomotic site further delay gastrointestinal function recovery and worsen intestinal narrowing. Consequently, gastric tube feeding becomes more challenging, as food stagnates in the dilated gastrointestinal tract and struggles to pass through the anastomosis into the distal bowel. This increases the risks of gastroesophageal reflux-related vomiting, aspiration, and anastomotic leakage (17, 18). In light of these challenges, we recommend prioritizing the use of a nasojejunal tube bypassing the duodenum for enteral nutrition in infants following duodenal atresia repair. Xiao et al. (19) demonstrated that the nasojejunal tube is not only simple to implement but also maintains mucosal integrity and reduces bacterial translocation. This approach can effectively enhance the patient's nutritional status, boost immune function, promote IGF-1 synthesis and secretion, mitigate postoperative inflammation, and reduce the stimulation of intestinal anastomosis by nutrition solution. Consequently, this method may lower the risk of postoperative complications and facilitate patient recovery.

The most common cause of CDO is HP, accounting for 74.3% of cases in a retrospective analysis of 105 children with CDO (20). In addition to HP, CDO is frequently associated with other congenital anomalies, including congenital heart disease, intestinal malrotation, genitourinary malformations, esophageal atresia, and Down syndrome (9). However, the co-occurrence of CDO and HP has been infrequently documented in the literature.

HP, also referred to as ectopic pancreas or accessory pancreas, is predominantly asymptomatic in most patients. It is frequently discovered incidentally during abdominal surgery. A retrospective analysis of 2,737 pediatric abdominal surgeries revealed an HP incidence of approximately 0.44% (21). HP is primarily located in the upper gastrointestinal tract, followed by the proximal jejunum (21, 22). In symptomatic patients, recurrent abdominal pain is the most common manifestation. Other presenting symptoms may include indigestion, frequent vomiting, gastrointestinal bleeding, melena, pancreatitis, intestinal intussusception, and gastrointestinal obstruction (23, 24).

The etiology of HP remains a subject of ongoing debate. One of the most widely accepted theories is the misplacement theory, which posits that during embryonic development of the pancreas and axial rotation of the primitive gut, a portion of the pancreatic tissue is displaced onto the developing foregut, ultimately forming an ectopic pancreas isolated from the normal pancreatic anatomy. This theory effectively explains why most HP tissue is found in the upper gastrointestinal tract (7, 25). Conversely, the metaplasia theory suggests that during embryonic development, endodermal tissues migrate to the submucosa and undergo pancreatic metaplasia, eventually developing into a HP. This theory helps elucidate why HP is also observed in organs of non-foregut origin (25).

The identification of HP lesions through radiological means is challenged by the absence of distinctive clinical symptoms and anatomical variations. In pediatric cases, the typically small size and asymptomatic nature of HP tissue in early stages further complicate diagnosis. Consequently, most instances are discovered incidentally during abdominal surgeries. The significant disparity between the intraoperative detection rate of approximately 0.44% and the autopsy-reported incidence of 0.5%–13.7% (4, 21) suggests a high probability of intraoperative diagnostic oversight. Several factors may contribute to this discrepancy, including: (1) limited clinician awareness of HP; (2) restricted field of view in open surgery; (3) absence of thorough abdominal exploration. In this particular case, preoperative ancillary examinations did not indicate the presence of a HP. Its detection was facilitated by the use of ICG fluorescence imaging, which enhanced the visibility of the HP within the intestinal wall.

While some instances of HP may not result in severe complications, failing to identify and address these ectopic tissues during abdominal surgery can increase the complexity and challenges of subsequent procedures, particularly due to adhesions in the abdominal cavity. Therefore, in cases such as this one, where the initial diagnosis is CDO, potentially accompanied by other congenital anomalies, it is crucial to thoroughly examine the abdominal organs during surgery to prevent underdiagnosis. Research has shown that HP carries a risk of malignant transformation into adenocarcinoma or follicular cell carcinoma (26, 27). Consequently, surgical resection is recommended for all cases of HP with clinical symptoms or incidental intraoperative findings to prevent potential complications and malignancy associated with HP.

In conclusion, the application of ICG molecular fluorescent imaging technology and the duodenal obstruction point suspension traction method in laparoscopic surgery for CDO significantly enhances the procedure's efficacy. These techniques not only reduce procedural complexity and improve success rates but also result in more aesthetically pleasing incisions with minimized surgical trauma. Postoperative use of the nasojejunal tube has been shown to accelerate the transition to enteral nutrition and mitigate postoperative complication risks, thereby shortening hospital stays. For surgical interventions involving conditions associated with concomitant congenital anomalies, a thorough abdominal exploration is essential to detect the presence of HP. When HP is identified during surgery, resection is recommended. This comprehensive approach ensures optimal patient outcomes and addresses potential concurrent malformations effectively.

## Data Availability

The raw data supporting the conclusions of this article will be made available by the authors, without undue reservation.

## Supplementary material

Video 1 Video of the laparoscopic surgery.
